# Emerging roles of H3K9me3, SETDB1 and SETDB2 in therapy-induced cellular reprogramming

**DOI:** 10.1186/s13148-019-0644-y

**Published:** 2019-03-08

**Authors:** Joachim Torrano, Abdullah Al Emran, Heinz Hammerlindl, Helmut Schaider

**Affiliations:** 1 The University of Queensland Diamantina Institute, University of Queensland, Brisbane, QLD Australia; 2 Centenary Institute of Cancer Medicine and Cell Biology, University of Sydney, Camperdown, NSW Australia

**Keywords:** SETDB1, SETDB2, H3K9me3, IFN signalling, Adaptive resistance, Transcriptional reprogramming

## Abstract

**Background:**

A multitude of recent studies has observed common epigenetic changes develop in tumour cells of multiple lineages following exposure to stresses such as hypoxia, chemotherapeutics, immunotherapy or targeted therapies. A significant increase in the transcriptionally repressive mark trimethylated H3K9 (H3K9me3) is becoming associated with treatment-resistant phenotypes suggesting upstream mechanisms may be a good target for therapy. We have reported that the increase in H3K9me3 is derived from the methyltransferases SETDB1 and SETDB2 following treatment in melanoma, lung, breast and colorectal cancer cell lines, as well as melanoma patient data. Other groups have observed a number of characteristics such as epigenetic remodelling, increased interferon signalling, cell cycle inhibition and apoptotic resistance that have also been reported by us suggesting these independent studies are investigating similar or identical phenomena.

**Main body:**

Firstly, this review introduces reports of therapy-induced reprogramming in cancer populations with highly similar slow-cycling phenotypes that suggest a role for both IFN signalling and epigenetic remodelling in the acquisition of drug tolerance. We then describe plausible connections between the type 1 IFN pathway, slow-cycling phenotypes and these epigenetic mechanisms before reviewing recent evidence on the roles of SETDB1 and SETDB2, alongside their product H3K9me3, in treatment-induced reprogramming and promotion of drug resistance. The potential mechanisms for the activation of SETDB1 and SETDB2 and how they might arise in treatment is also discussed mechanistically, with a focus on their putative induction by inflammatory signalling. Moreover, we theorise their timely role in attenuating inflammation after their activation in order to promote a more resilient phenotype through homeostatic coordination of H3K9me3. We also examine the relatively uncharacterized functions of SETDB2 with some comparison to the more well-known qualities of SETDB1. Finally, an emerging overall mechanism for the epigenetic maintenance of this transient phenotype is outlined by summarising the collective literature herein.

**Conclusion:**

A number of converging phenotypes outline a stress-responsive mechanism for SETDB1 and SETDB2 activation and subsequent increased survival, providing novel insights into epigenetic biology. A clearer understanding of how SETDB1/2-mediated transcriptional reprogramming can subvert treatment responses will be invaluable in improving length and efficacy of modern therapies.

## Background

The expected impact of modern therapies has diminished with laboratory and clinical findings that prolonged exposure to these treatments tends to cultivate insensitivity and resistance in the surviving fraction of tumour cells. Cell-to-cell variation amongst tumour populations is a well-established characteristic of cancer and this heterogeneity allows for the selection and development of subpopulations with intrinsic and acquired resistance mechanisms to current treatments [1–4].

Current standards of care such as conventional chemotherapeutics, T cell therapy, immune checkpoint inhibitors and targeted therapies are limited by the development of resistance in tumours that originally displayed a significant initial response [5–8]. While this can be attributable to mutations in genes affecting proliferation, apoptosis, cell efflux and other important biological processes, accumulating reports now show, even without mutation, tumour cells can survive drug exposure through significant yet transient changes in their phenotype that lead to acquired resistance [8–11].

The development of these acquired resistance mechanisms across different treatments show similarities in the alterations to their transcriptional profile, signalling pathways and chromatin structure prior to phenotypic reversion [4, 5, 8, 12–15]. A new understanding is growing from recent studies that drug exposure—or perhaps any significant cytotoxic stress—encourages epigenetic “switching” that promotes a transient, slow-cycling phenotype resistant to apoptosis [2, 16, 17]. Currently, these phenotypes are most well studied in melanoma and lung cancer cell lines reported to develop acquired resistance to targeted therapy which appears to be characterised by inducible and reversible enrichment of interferon (IFN) pathway signalling [6, 7, 13], cell cycle inhibition [16, 18, 19] and transcriptional repression of a subset of genes due to increased repressive chromatin modifications such as trimethylation of histone 3 lysine 9 (H3K9me3) [14, 15]. Many recent reports have been investigating the development of adaptive drug resistance in cancer, and it is becoming increasingly apparent that epigenetically mediated reversible transition into a slow-cycling state marked by repressed transcriptional activity is a common key feature [5, 12]. Amongst other features, increased H3K9me3 seems to be one of the major regulators of this slow-cycling phenotype [14, 15] together with upstream methyltransferases SETDB1 and SETDB2. Hereafter, we will discuss general characteristics of therapy-induced reprogramming in cancer phenotypes with a focus on IFN signalling and epigenetic remodelling, before focusing on the H3K9me3-specific histone methyltransferases SETDB1 and SETDB2 and their detailed role (s) in biology and the development of adaptive drug-tolerance in these aforementioned phenotypes.

## Therapy-induced reprogramming achieves adaptive drug-resistance

### A common phenotypic transition in treated tumour populations marked by sequential and shared transcriptomic alterations

The development of acquired resistance following treatment is not always through the selection of inherently resilient subpopulations or the acquisition of de novo mutations, as evidenced in a seminal paper by Sharma et al. showing reversible resistance in single-cell-derived clones [8]. Instead, it is becoming more apparent that initial drug tolerance and eventual acquired resistance are characteristics potentially gained through phenotypic switching to a transiently reprogrammed state occurring in treated populations [4]. The drug-tolerant persister (DTP) state originally described in EGFR mutant non-small-cell lung cancer (NSCLC) cells by Sharma et al. [8] has been further investigated in a series of follow-up papers: our lab has shown a highly similar phenotypic transition to a drug-tolerant state (termed-induced drug-tolerant cells or IDTCs) using BRAF and NRAS mutant melanoma exposed to BRAF inhibitors, hypoxia or nutrient starvation [5, 15]. Our lab and Sharma et al. have both observed loss of the transcriptionally active histone mark H3K4me3 and increases in H3K4me3 demethylases, KDM5A and KDM5B respectively, indicating an H3K4me3-reducing epigenetic mechanism responsive to environmental challenge by treatment or hypoxia [5, 8]. Sharma et al. also observed increased cytotoxicity to combination treatment with HDAC inhibitors, although we found that HDAC inhibitor treatment did not reverse the H3K4me3 loss or H3K9me3 gain consistently seen in IDTCs, suggesting a robustness in maintaining epigenetic changes inherent in the phenotypic transition [5, 8].

A publication by Hugo et al. analysed whole-exome sequences from MAPK inhibitor-resistant melanoma biopsies and found 39% did not contain a known resistance-mediating mutation indicating the potential prevalence of non-genetic resistant phenotypes [20]. This study indicated DNA methylation rather than histone modifications as the principal regulator of the transcriptome in drug-resistant cells, although crosstalk between the two epigenetic mechanisms does exist considering the presence of methyl-binding domains in SETDB1 and SETDB2 allowing interaction with DNA methyltransferases [20, 21]. Activating epigenetic alterations for resistant cells typically occur at resistance-mediating genes such as *Wnt5A*, *FGFR*, *AXL*, *EGFR*, *NGFR* and *JUN*, and interestingly Shaffer et al. found these genes are often co-expressed in individual cells due to cellular reprogramming and chromatin restructuring identified by ATAC-seq (Assay for Transposase-Accessible Chromatin using sequencing) [12]. Shaffer et al. analysed transcriptomics and single-cell RNA fluorescent in situ hybridisation (FISH) data from the lung, breast, cervical and melanoma cell lines and primary melanocytes finding that expression of resistance-mediating genes was highly variable amongst populations, yet sporadically coordinated together in rare individual cells. Treating melanoma populations with a BRAF inhibitor promoted greater survival of the rare cells and a transition to a common transcriptional profile brimming with activated genes of different resistance-mediating pathways such as the ones listed above [12]. Shaffer et al. also noted rare-cell expression in non-cancerous melanocytes and suggested this mechanism likely extends to other cell types. The expression of AXL, EGFR and NGFR in melanoma has also been reported as markers for epithelial-mesenchymal transition and dedifferentiation indicating a loss of the original phenotype [22–24]. Logically, these rare resistant cells identified in Shaffer et al. possess a clear fitness advantage over non-resistant cells during exposure to treatments and could represent an early stage of the DTP/IDTC phenotype, which is supported by increased levels of NGFR in IDTCs [5].

Therapy-induced transcriptional reprogramming was also observed in Song et al. who compared transcript profiles from regressing patient samples, treated melanoma cell lines and treated murine melanomas to pre-treated controls finding that surviving melanoma cells have transcriptional reprogramming trajectories that cluster towards either a MAPK-reactivated state or a MAPK-independent state [13]. Mitogenic rewiring of treated melanoma cells to achieve pathway reactivation or bypass appears vital for resistance to targeted therapy as shown by systems biology analysis [25]. Moreover, the MAPK-reactivated cells in Song et al. had a transcriptome similar to untreated cells indicating a re-establishment of the original phenotype with reduced sensitivity to treatment, whereas the MAPK-independent cells were transcriptionally distinct [13]. Intriguingly, melanoma cell line-derived DTPs displayed a similar transcriptomic trajectory to MAPK-independent populations suggesting converging phenotypes as well as other notable characteristics: morphological flattening alongside a proposed mesenchymal-angiogenic switch, initial increases in β-galactosidase as a sign of senescence and heterochromatin formation, and lastly enrichment of inflammatory and IFN pathway signalling [13]. Many of these qualities were also observed for subpopulations of NSCLC-derived DTPs as well as IDTCs from melanoma, breast, lung, colorectal and liver cancer [5, 6, 8, 13–15], suggesting these converging phenotypes are representative of a broadly applicable mechanism. A summary of shared characteristics in studies of resistant cancer cells is provided in Table 1.

**Table 1:** Studies of treatment-induced resistant phenotypes and common characteristics

Model(s) used	Treatment method(s)	Treatment duration	Characteristics observed in surviving cells	Reference
Non-small-cell lung cancer (PC9, HCC827), melanoma (M14), colorectal cancer (Colo-205), breast cancer (MDA-MB175v2, SKBR3, HCC1419), gastric cancer (KATO II)	Erlotinib (2 μM), AZ628 (2 μM), Lapatinib (2 μM), PF-2341066 (1 μM)	9 days, long-term assays up to > 30 days	Phenotypic switching, drug insensitivity, mitogenic rewiring, global histone alterations	[8]
Melanoma (11 BRAF mutant, 3 BRAF wild type), in vitro and in nude mice	Vemurafenib (0 .5μM)	7 days	G1 arrest, increased senescence markers, phenotypic switching, heterochromatin formation	[40]
Melanoma (3 BRAF mutant, 1 NRAS mutant)	Vemurafenib (250/500 nM), cisplatin (30 μM), low glucose media, hypoxia	> 12 days, long-term assays up to 75 days	Phenotypic switching, multi-drug insensitivity, dedifferentiation, increased angiogenic and tumorigenic potential, global histone alterations	[5]
90 biopsies from melanoma patients pre- and on-treatment with disease progression	MAPK-Targeted therapy (i.e. BRAF inhibitors, BRAF + MEK inhibitors)	–	Highly recurrent transcriptomic alteration, mitogenic rewiring, increased resistance to both targeted therapy and immune checkpoint inhibitors	[20]
Breast cancer (TSA) and melanoma (B16-F10) cells in vitro or implanted in mice	Immune checkpoint inhibitors (anti-PD1, anti-CTLA4), type 1 and 2 IFNs	< 16 days	Increased IFN signalling, cross-resistance to anti-CTLA4 therapy via T cell receptor depletion, epigenomic alterations	[6]
Leukaemia (L1210)	Chemotherapeutics (Carmustine 2 .5 μg/mL, Vincristine 10 ng/mL, cytarabine 1 μg/mL)	18–24 h	Increased drug resistance, survival significantly associated with reduced proliferation	[16]
Non-small-cell lung cancer (PC9) and single-cell-derived subpopulations (PC9–1)	Erlotinib (2 .5 μM), WZ8040 (0 .1 μM), WZ3146 (0 .1 μM), SGX-523 (0 .1 μM), Crizotinib (31.6 nM), trichostatin A (20 nM)	14 days, long-term assays up to > 46 weeks	Increased drug resistance, growth arrest, diverse mechanisms of gaining de novo resistance	[2]
Non-small-cell lung cancer (PC9), colorectal cancer (SW480, Colo205), breast cancer (SKBR3, EVSAT), melanoma (M14, Hs888, C32), gastric cancer (GTL-16)	Erlotinib (1 μM), GDC-0980 (2 μM), AZ628 (2 μM), Lapatinib (1 μM), Vemurafenib (2 μM), 5-FU (33 μM), SN-38 (6 nM), crizotinib (1 μM)	7–28 days depending on cell line and treatment	Increased drug resistance, growth arrest, global histone alterations, retroviral activation and subsequent repression by H3K9me3	[14]
Melanoma (WM989, WM983B, 1205Lu, SK-MEL-28), primary melanocytes	Lapatinib (1 μM), Vemurafenib (1 μM),	2–28 days	Dedifferentiation, epigenetic reprogramming, rare co-expression of resistance genes,	[12]
46 patient-matched melanomas pre- and on-treatment, 7 melanoma cell lines + murine melanoma in nude mice	MAPK-targeted therapy (i.e. BRAF inhibitors, BRAF + MEK inhibitors)	–	Highly recurrent transcriptomic alteration, increased mesenchymal and angiogenic potential, increased IFN signalling, decreased immune sensitivity	[13]
Melanoma (WM164, WM1366), lung cancer (A549, HCC827), colon cancer (HT29), liver cancer (HEPG2), breast cancer (SKBR3)	Dabrafenib (25 nM), Trametinib (10 nM), Erlotinib (5 μM), docetaxel (5 nM/30 nM), Doxorubicin (500 nM), cisplatin (80 nM), low glucose media	12–15 days	Phenotypic switching, multi-drug insensitivity, global histone alterations, enriched IFN signalling	[15]
Case study of metastatic melanoma patient, 8 melanoma cell lines	Adoptive T cell therapy, TNFa supplementation	–	Increased immunotherapy resistance, reversible and inflammation-induced dedifferentiation	[7]
4 patient-derived primary B cell lymphomas, haematological malignancies (RCK8, EHEB, K562, Mec1), colorectal cancer (SW480, LS174T, DLD-1, Caco-2), melanoma (WM266.4, SK-Mel-28, MeWo, Omm 2.3)	Adriamycin (0.01–0.05 μM), ICG-001 (1 μM), Salinomycin (1 μM), PD325901 (10 nM), PD98059 (25 μM), LY294002 (10 μM), MK-2206 (200 nM), CHIR99021 (1 μM)	2–7 days	Dedifferentiation, phenotypic switching, increased drug resistance, increased tumorigenic potential, temporary senescence features, heterochromatin formation	[28]

### Therapy-induced senescence leads to a common, dedifferentiated, slow-cycling and drug-resistant transcriptional state

Activating mutations in growth pathways initially trigger rapid cell proliferation until the onset of a cell-autonomous mechanism to prevent DNA damage from accelerated divisions previously termed oncogene-induced senescence [26]. Benign growths such as melanocytic nevi have been found to contain hyperactive BRAF mutations similar to melanomas but do not undergo tumorigenesis due to the maintenance of the senescent state [27]. Senescence was originally characterised by increased senescence-associated β-Galactosidase, increased heterochromatic foci and H3K9me3, a distinct secretory phenotype, reduced apoptotic signalling and irreversible cell cycle arrest [26]. New research has shown that when cancer populations are exposed to treatments (i.e. chemotherapy, targeted therapy), they enter a senescent-like state that has been termed ‘therapy-induced senescence’ that is capable of both protecting cancer cells and reactivating proliferation with increased growth and invasive potential [28]. Additionally, H3K9 demethylases from the two structurally unrelated LSD and JMJ families have been shown to be responsible for promoting transcription of proliferative E2F target genes in melanoma in order to reactivate the cell cycle, supporting the idea that H3K9 methylation is an important aspect of maintaining this senescence-like, drug-resistant state [29]. Therapy-induced alterations to tumour secretomes have also been found to promote resistance and tumour progression in melanoma and lung cancer, further suggesting the involvement of the tumour microenvironment and paracrine signalling potentially mediated by this senescence-like state [30].

This process of cell cycle arrest is promoted through the accumulating activity of autocrine/paracrine type I interferons (IFNα/β) that are continuously induced and secreted by cells in response to DNA damage in an ATM-IKKα/β-IRF3-dependent manner [31]. A hyperactive MAPK pathway bypasses this IFN-mediated senescence mechanism through BRAF-mediated activation of SCF-βTrcp2/HOS E3 ligases that ubiquitinate and degrade the type I IFN receptor IFNAR1 [32]. Katlinskaya et al. showed that treatment with BRAF inhibitors in melanoma allows re-expression of IFNAR1 and subsequently restores type I IFN signalling [33]. Other cancer types such as colorectal, ovarian and lung cancers also occasionally (less than 10% of cases) exhibit activating BRAF mutations that in turn stimulate SCF-βTrcp2/HOS ligase activity that ubiquitinates several tumour suppressors (often cell cycle inhibitors or cytokines) and promotes tumour progression [34]. For more detail, these dysregulated proteins are covered in a review by Frescas and Pagano [34].

The relationship between senescence signalling and BRAF inhibitor resistance is best exemplified in melanoma where drug resistance is also typically accompanied by dedifferentiation markers such as NGFR, which is inducible by type II interferons (IFNγ) [5, 35, 36]. While IFNα/β and IFNγ are distinct in their receptors, activators and downstream effects, they do experience crosstalk through their mutual regulation of STAT1 phosphorylation, a transcription factor capable of inducing SETDB2, NGFR, p53 and other factors in melanoma dedifferentiation and senescence [37–39]. Many features of senescent cells are shared with drug-treated tumour cells from other studies: chemo-resistance, low metabolism, phenotypic flattening, increased heterochromatin formation and increases in the p53 protein [17, 26, 40–42]. This increase in heterochromatin is reflective of a condensed genomic structure via increased histone methyltransferases and elevated levels of senescence-associated heterochromatin foci, accompanied by increased recruitment of the retinoblastoma (Rb) protein and H3K9me3 at *E2F* promoters to prevent cell cycle progression [43]. Hence, cell cycle arrest and heterochromatin formation occur in both treated tumour cells and senescent cells, alongside resistance to conventional and new-generation treatments such as taxanes, anthracyclines and targeted therapies [42].

The role of IFNs in adaptive resistance extends to immune checkpoint blockade (ICB), as evidenced by Benci et al. who reported that prolonged IFN signalling in melanoma cells promotes epigenetic restructuring to match cells resistant to anti-PD1 immunotherapy through transcriptional regulation of multiple T cell inhibitory receptors [6]. Although this effect was only seen with extended IFNγ treatment over 2–3 weeks, both types I and II IFN signalling contributed to the maintenance of this resistant phenotype [6]. ATAC-seq of CD45-negative sorted melanoma cells also showed that 45.9% of open chromatin regions acquired by an IFN-treated, ICB-resistant melanoma cell line overlapped with acquired open chromatin regions in samples derived from patients with relapsing tumours that initially responded well to immunotherapy, suggesting a common epigenetic mechanism [6]. These open chromatin regions were also found to display high levels of STAT1 binding motifs and STAT1 occupancy indicating that one important outcome of this epigenetic restructuring was to enrich IFN signalling through the upregulation of STAT1 target genes [6]. The relationship between epigenetic dysregulation and senescence in cancer seems to be multifaceted and is another blossoming subject of research that is covered in a review by Decottignies and Fagagna [44].

### Epigenetic rewiring via therapy-induced IFNs may confer reversible, convergent and drug-resistant phenotypes across different cancer populations

Phenotypic switching, increased senescence signalling and IFN enrichment observed in DTP melanoma cells by Song et al. were also features reported in our IDTCs models derived from melanoma, breast and lung cancer-derived cell lines [15]. It should also be noted that the transcriptional reprogramming observed in Song et al. was characterised by repression of proliferative and invasive genes via differential DNA methylation and H3K27 acetylation (H3K27ac) and that we detected slightly increased global DNA methylation and loss of H3K27 tri-methylation (H3K27me3) in melanoma IDTCs compared to untreated cells [13, 15].

H3K27 methylation and acetylation are mutually antagonistic; therefore, a decrease in H3K27me3 levels could be reflective of increased H3K27ac [45]. Additionally, activated Akt can phosphorylate EZH2 (a H3K27me3 methyltransferase) and thereby suppress its activity [46]. We have reported increased levels of Akt signalling in IDTCs which may explain the accompanying decrease of H3K27me3 [5]. These similarities in histone modifications between treatment-induced resistant cells of these studies support the notion that treated melanoma cells transition to a resistant phenotype via epigenetically mediated transcriptional reprogramming.

The studies above identify common characteristics of resistant phenotypes in melanoma and other cancers, which include dedifferentiation, mitogenic rewiring, chromatin restructuring and inflammatory signalling especially via IFN pathway enrichment. Additionally, melanoma cells resistant to treatment appear to progress through distinct states of reversibility, epigenetic regulation, dedifferentiation and proliferation at different times indicating that the development of adaptive resistance follows a stepwise progression [4, 8, 11]. Sharma et al. showed that continuous treatment of DTPs with erlotinib for over 30 days saw the rise of DTEP/DTPPs (drug-tolerant expanded/proliferating persisters, used interchangeably in these studies) that remain resistant to treatment, progress through the cell cycle at a much faster rate than DTPs and lose expression of dedifferentiation markers [8]. Multi-stage dedifferentiation in melanoma cell lines exposed to MAPK inhibitors or inflammatory signalling was also denoted in Tsoi et al., where they performed principal component analysis of transcriptional profiles from resistant melanomas to find a transcriptomic trajectory along four distinct clusters that matched sequential progression of human embryonic stem cells into neural crest, melanoblast and melanocyte stages [11]. This dedifferentiation occurs as early as 3 days into treatment with IFNγ or another inflammatory cytokine TNFα, suggesting that without drug exposure, inflammatory signalling can drive this particular phenotypic transition [11].

IFN enrichment as a characteristic of resistant cancer cells may appear paradoxical considering its frequent use in combination treatments for antiproliferative effects on early-stage melanomas and other tumours [47]. Only IFNβ is expressed in untreated melanoma cells; however, levels of secreted IFNγ and TNFα become significantly increased in the sera of melanoma patients under targeted therapy [48, 49]. Increased inflammatory signalling leading to resistance has been reported for both targeted therapies and immunotherapies (e.g. MAPK and immune checkpoint inhibitors respectively) and the ability of IFNs to promote dedifferentiation in melanoma is well established [6, 15, 50]. Aberrant DNA hypomethylation and dysregulated chromatin factors partially activated by IFNγ are associated with increased PD-L1 in melanoma promoting resistance to immune checkpoint blockade highlighting the ability of epigenetic mechanisms to encourage resistant phenotypes [51].

Increasing TNFα has been shown to promote reversible melanoma dedifferentiation through c-Jun-mediated suppression of the melanocytic differentiation marker MITF and can confer resistance to T cell therapy [50, 52]. Similarly, targeted therapy has been found to boost type I IFN signalling that promotes cell cycle arrest and dedifferentiation in BRAF mutant melanoma [33]. The extent to which inflammatory cytokines, in particular IFN signalling, can induce phenotypic changes that contribute to therapeutic resistance has not been extensively studied. However, the following section will address recent reports highlighting the consistent enrichment of inflammatory signalling at the onset of treatment [6, 13–15] as well as its potential to induce dedifferentiation, cell cycle arrest and the histone methyltransferases SETDB1 and SETDB2.

### IFN signalling, SETDB1/2 induction and chromatin remodelling as potential drivers of phenotypic switching and therapeutic resistance

Similar to Emran et al. and Song et al., another recent study by Guler et al. also observed IFN pathway enrichment in DTPs derived from the EGFR mutant NSCLC cell line PC9 [14]. The IFN pathway is responsive to DNA damage caused by cancer treatments and the activation of downstream interferon regulatory factor (IRF) genes can potentiate cell survival [53]. Several IFN response/antiviral defence markers such as *IFI6*, *IFI16*, *IFIH1*, *TLR3* and the key IFN regulator *IRF7* were amongst the most induced genes in DTPs [14]. Treatment of PC9 cells with the EGFR inhibitor erlotinib was found to promote chromatin accessibility at the promoters of the above genes as identified through ATAC-seq [14]. This increase in chromatin accessibility at specific genes occurred alongside a regional increase in the repressive, chromatin-condensing marks H3K9me3 and H3K27me3 over endogenous retroviral elements (ERVs) such as LINE-1 [14]. They also performed an analysis of tumour biopsies from patients prior to and during vemurafenib (BRAFi) treatment finding high SETDB1 expression in on-treatment tumours [14]. SETDB1 has been shown in another study of acute myeloid leukaemia to silence ERVs and inhibit IFNs, which may be a crucial step in developing resistance to treatment [54]. H3K9me3-ChIP-seq of DTPs by Guler et al. showed distinct regional H3K9me3 enrichment consistent with Emran et al., and further analysis of H3K9me3 distribution at repetitive regions showed a significant increase of H3K9me3 over LINE-1 retrotransposons [14, 15]. Disruption of H3K9me3 over LINE-1 retrotransposons by either HDAC inhibitors or the stable knockout of SETDB1/G9a could be a possible way to eliminate these DTPs observed with upregulation of LINE-1 [14], indicated in another paper showing that induction of ERVs correlated with DNA methyltransferase inhibitors enhancing the effectiveness of immune checkpoint inhibitor therapy [55].

In addition to their ability to induce DNA double-stranded breaks, studies in multiple cell types have shown LINE endogenous retroelements are capable of activating viral sensors reactive to dsRNA such as Rig-1 and TLR3 and subsequently stimulating inflammatory response genes including the IFN pathway to promote cell cycle arrest and apoptosis [56–58]. Moreover, a stress-responsive alternative kinase of the MAPK pathway, p38, activates following environmental challenges such as hypoxia or drug exposure and can subsequently induce LINE-1 expression [59]. Lastly, senescent cells have been found to undergo epigenomic alterations associated with the induction of active, ‘young’ ERVs that are repressed via DNA methylation, in contrast to more ancient, defunct ERVs that are repressed by histone methylation [60, 61]. Keeping in mind the limited amount of specific data available, SETDB1 and SETDB2 are H3K9me3-methyltransferases with methyl-binding domains and established links to IFN signalling, and therefore it is probable that one or both of them are responsible for the silencing of drug-induced cytotoxic ERVs and IFNs following treatment.

## SETDB1 and SETDB2: emerging mediators of inducible, drug-resistant phenotypes

### SETDB1/2 structure and function: H3K9 trimethylation via the SET domain

SETDB1 and SETDB2 are members of the SUV39 family of lysine methyltransferases, which also include SUV39H1, SUV39H2, G9a, GLP, SETMAR and SETD8 [62]. They typically share pre- and post-SET domains rich in cysteines and flanking a central SET domain responsible for catalysing the separation of a methyl group donated from S-adenosylmethionine—a stable metabolite important for transmethylation, transsulfuration and polyamine synthesis pathways—and depositing the methyl group onto a lysine residue of a target protein such as a histone [63]. Unlike other family members, SETDB1 and SETDB2 contain a methyl-CpG-binding domain, and this has been shown for SETDB1 to allow recruitment to methylated CpG islands to deposit H3K9me3 at nearby histones [64]. This stimulates local heterochromatin formation to complement DNA methylation and is suggested to stratify the level of transcriptional repression at specific loci to more precisely control gene expression [64]. Due to high sequence similarity, this function may also extend to SETDB2.

**Fig. 1:**
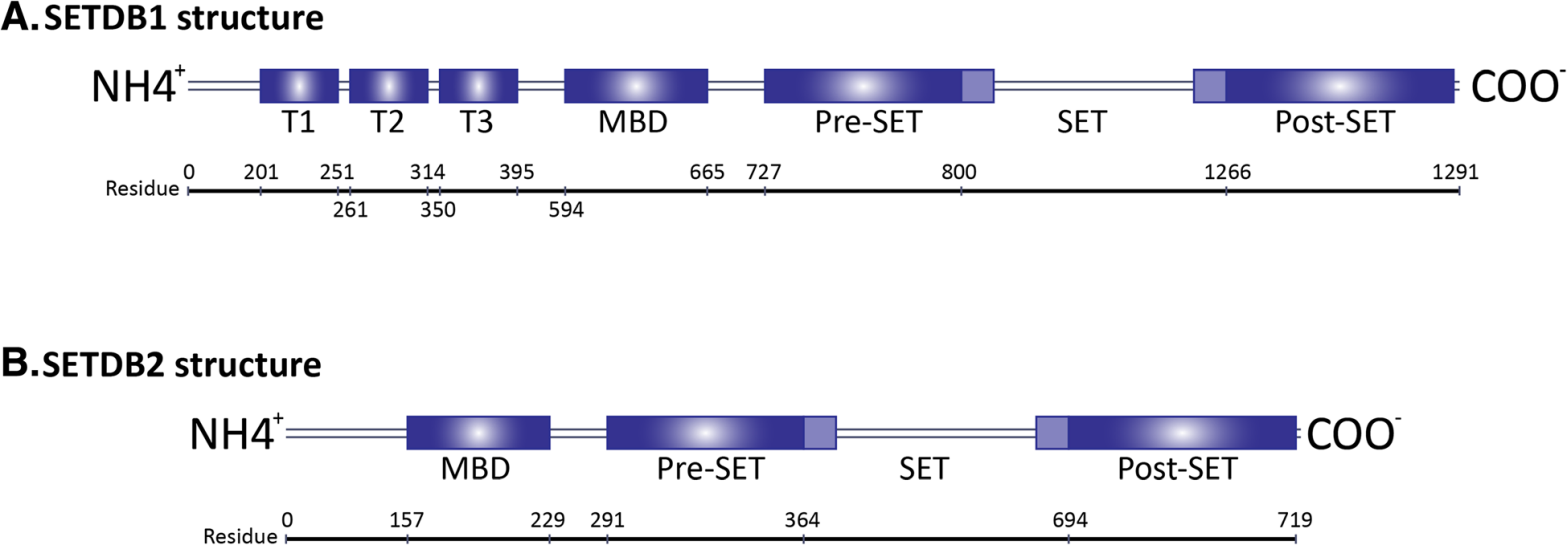
Diagrammatic map of the SETDB1 (**a**) and SETDB2 (**b**) proteins. Illustration of the residual positions of the triple Tudor domain (T1, T2 and T3), methyl-CpG binding domains (MBD) and bifurcated SET multidomains

Moreover, while SUV39H1/2-mediated H3K9me3 is limited to heterochromatin, SETDB1 is active in euchromatic regions similar to H3K9me1/2-methyltransferases G9a and GLP implying that SETDB1 is more involved in gene regulation than SUV39H1/2 [65]. Currently, the chromatin-specific localization of SETDB2 has not yet been investigated. SETDB1 and SETDB2 are also distinct from other SUV39 proteins in that they share a hugely bifurcated SET domain with insertion sequences of 73% homology indicating that they are derived from a protein with the same insertion, although the SETDB1 insert comprises 347 amino acid residues whereas the SETDB2 insert is only 218 [66]. SETDB1 also possesses a triple Tudor domain that is reported to bind and remove acetylated H3K14 through KAP1-mediated recruitment of the NuRD/HDAC complex to further the condensation of chromatin [67, 68]. SETDB1 and SETDB2 gene information are shown in Fig. 1.

The SET domain is highly conserved amongst eukaryotes, appearing in homologues from other animals, plants, fungi and yeast [63]. The crystallisation of these variant SET domains characterised its conformational folding as a turn and loop-rich structure with two distinct subdomains comprising antiparallel β-strands separated by a highly variable segment [62]. This segment is usually quite short in most methyltransferases; however, it does coincide with the bifurcating sequences in SETDB1 and SETDB2, as well as insertions of entire protein domains in other SET-domain proteins such as Rubisco, LSMT and the SMYD family of enzymes [69]. The > 200 residue bifurcating insertions in the SETDB1 and SETDB2 proteins may therefore be vestigial sequences of no relevance to their methyltransferase function but could be nonetheless important for other roles. The SET domain appears to retain its methyltransferase ability regardless of the distance between subdomains and this feature may allow for greater structural variation and complexity in interactions with histones, their most common substrate.

Histone methylation at H3K9 by SET domain-containing proteins is vital during development and an important mediator of heterochromatin formation, gene silencing and genomic stability [70]. Epigenetic modifiers of H3K9 are highly conserved throughout metazoan genomes illustrating their significance in regulating chromatin architecture and transcription during cell and tissue development [71]. In cancer, dysregulation of H3K9me3 via aberrant SETDB1 expression can silence tumour suppressor genes such as *APOE*, *p53* and *HoxA*, and promote a more aggressive tumour phenotype in melanoma, ovarian, lung, liver and breast cancers [72]. Conversely, SETDB1 has also been found to silence the oncogene ANXA2 to achieve tumour-suppressor roles such as suppressing distal metastasis in lung cancers [73]. These paradoxical findings suggest that SETDB1 can act as a proto-oncogene or tumour suppressor depending on the cellular context. Moreover, recent publications have shown that Akt and SETDB1 not only have the ability to directly interact [74], whereby SETDB1 promotes Akt signalling and directly represses pro-apoptotic gene transcription [75], but inducing this interaction also showed increased tumorigenesis in both NSCLC cell lines and mice models [76, 77].

SETDB1’s relationship with Akt does establish that SETDB1 is able to directly act on membranous and cytoplasmic components as well as downstream genes and significantly affect the general phenotype, highlighting the multifaceted nature of its activity. SETDB1’s role in development and tumourigenesis has recently been reviewed by Karanth et al. [72]; however, this review will discuss its role in reshaping the epigenome upon stress-induced induction of interferon signalling which remains unclear. Additionally, the potential mechanisms by which SETDB2 can exert the same multifaceted effects on cancer development have not been explored and will be the major focus of the following chapter.

### SETDB2’s roles in immunology, metabolism and development

SETDB2 is a relatively uncharacterised H3K9me3 methyltransferase that is only beginning to be a subject of research and so the following section briefly describes reports of SETDB2 to date. The majority of studies investigating the function of SETDB2 have often focused on general developmental biology, while its role in cancer is very much an emerging field. SETDB2’s neighbouring gene *PHF11* encodes a PHD zinc finger protein and the possible run-through transcription of PHF11 permits the generation of chimeric SETDB2-PHF11 mRNA with combined exons resulting in a fused protein [78]. SETDB2-PHF11 protein is calculated to weigh 98 kDa and its methyltransferase ability has not yet been tested. The frequent genetic dysregulation of the SETDB2-PHF11 transcript in asthma and atopic disease, alongside significant association with increased serum IgE levels, indicated a role in the immune response and specific polymorphisms of both genes have been classified as susceptibility markers for asthma and atopy [79–81].

Recently this immunological function was further elucidated in a report that SETDB2 is induced by the transcription factor STAT1 in response to type I interferon (IFNα/β) signalling to suppress the expression of antibacterial NFkB target genes and attenuate the inflammatory response [38]. Additionally, it has been shown that glucocorticoids signal through the glucocorticoid receptor (GR) to induce SETDB2 and promote GR-SETDB2 binding in murine liver cells leading to upregulation of the SREBP inhibitor *Insig2a* and increased lipogenesis [82]. This study demonstrates both a metabolic regulatory role for SETDB2 and the paradoxical upregulation of SETDB2 targets via promoter-enhancer bridging interactions.

Early research into SETDB2’s functions in zebrafish illustrated its crucial roles in embryonic development through timely H3K9me3-mediated repression of *fgf8*—to control dorsal organiser formation and left-right asymmetry—and *dvr1* to control convergence and extension movements during gastrulation [83, 84]. These findings would likely reflect SETDB2’s embryogenic roles in humans considering that 70% of human genes share orthologues in zebrafish and other species [85]. Additionally, recent studies have extended SETDB2’s role to mediating laterality not just at the cellular but at the organism level—the single nucleotide polymorphism *rs4942830* located in SETDB2’s first intron was found to be significantly associated with a person’s handedness (whether they are left or right-handed) establishing a link between atopy and handedness [86].

Studies of SETDB2 also document its role in stimulating cell proliferation, especially in cancer. A study this year showed it has been found to promote hyperproliferation and oncogenesis in acute lymphoblastic leukaemia by transcriptionally suppressing the cell cycle inhibitor CDKN2C [87]. This draws parallels to a study that showed SETDB1 expression is associated with CDKN2A repression in melanoma, suggesting that SETDB1 and SETDB2 have an important role in controlling the expression of these cell cycle regulators [88]. Knockdown of SETDB2 in human embryonic kidney (293 T) and epithelial cervix adenocarcinoma (HeLa) cell lines led to prolonged mitosis, abnormal spindle formation and loss of H3K9me3 as well as CENP B and C proteins (centromere components) at pericentric regions displaying an important role for SETDB2 in chromosomal segregation prior to metaphase [66]. This finding is complementary to a study of KDM4C, a lysine demethylase that significantly depletes mitotic H3K9me3—and dysregulation of this demethylase activity in osteosarcoma cells also causes abnormal mitotic phenotypes such as misaligned and lagging chromosomes during anaphase and telophase [89].

**Fig. 2:**
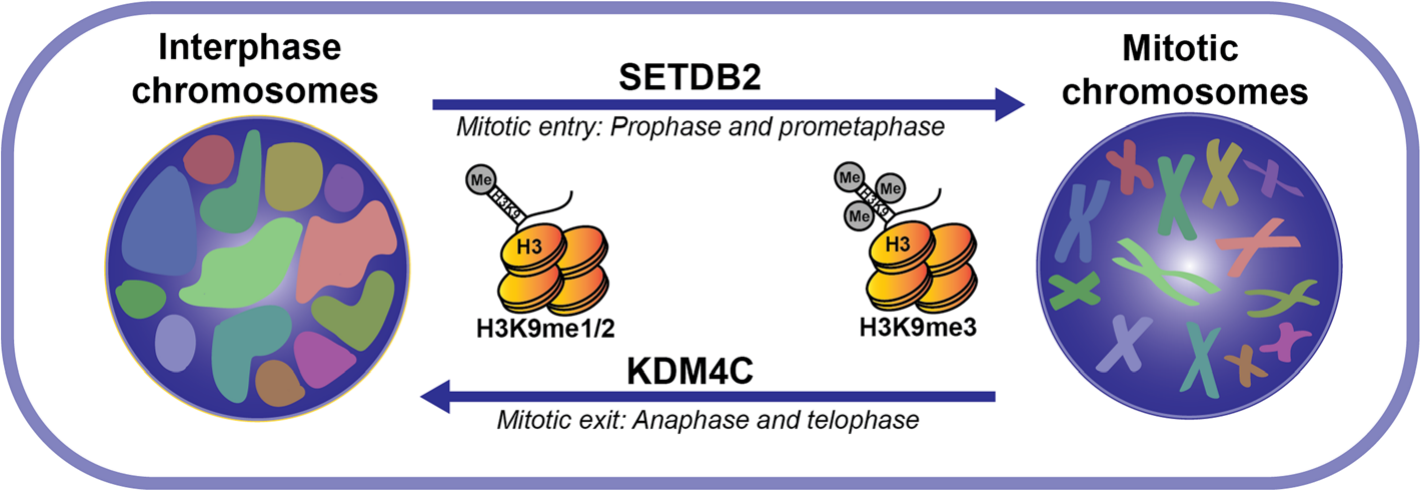
A simplified diagram of mitotic chromosomal remodelling by SETDB2. Model for putative chromosomal condensation and segregation during mitosis that is substantially contributed to via coordinated H3K9 methylation by SETDB2 and KDM4C. Other factors also contribute to this mechanism

These two studies above by Falandry et al. and Kupershmit et al. indicate that H3K9me3 levels are tightly coordinated by both SETDB2 and KDM4C during mitosis, and this regulation may be necessary for the conversion of chromosomal structures between tightly condensed mitotic chromatids and the looser chromosomal territories of interphase (Fig. 2). It is therefore unsurprising that the dysregulation of SETDB2 leads to increased genomic instability and has been associated with the development of multiple cancers.

### Aberrant SETDB2 expression in cancer and an emerging role in drug resistance

In contrast to family members SUV39H1/2 and G9a, SETDB2 is relatively unexplored and remains poorly characterised in cancer. SETDB2’s potentially exclusive function to preserve chromosome fidelity in mitosis may explain the finding that, unlike other methyltransferases including SETDB1, it is frequently downregulated in breast cancer [90]. Additionally, a recent study found low SETDB2 expression was associated with metastatic spread of late-stage renal cell tumours further suggesting a tumour-suppressive role [91]. On the other hand, SETDB2 overexpression is associated with poor prognoses and tumour progression in gastric cancer through H3K9me3-mediated silencing of tumour suppressor genes *WWOX* and *CADM1* [92]. An earlier study revealed that deletion of a 1 Mb gene cluster (48.2–49.2 Mb) of SETDB2 is significantly associated with chronic lymphocytic leukaemia progression [93]. In parallel, another study identified that a frameshift mutation within the mononucleotide repeat of the SETDB2 coding sequence is associated with microsatellite instability (MSI) in colorectal cancer [94]. This SETDB2 frameshift mutation was significantly higher in the MSI-high region of colorectal cancer compared to MSI-low regions, likely due to disruption of SETDB2’s important roles in maintaining genomic fidelity described above [94]. These reports illustrate that dysregulation of SETDB2 is a common step for tumorigenic progression in some cancers, summarised in Table 2.

**Table 2:** Reports of SETDB2 and its role in different cell types

Cell type(s)	Reported aberration of SETDB2	Regulatory role of SETDB2	Downstream target genes	Biological effects of SETDB2 activity	Reference
Acute lymphoblastic leukaemia	Upregulation	Inhibition	CDKN2C	Hyperproliferation	[87]
Gastric cancer	Upregulation	Inhibition	WWOX and CADM1	Apoptotic inhibition/may promote metastasis	[92]
Colorectal and gastric cancer	Frameshift mutation	–	–	Increased microsatellite instability	[94]
Renal cell carcinoma	Downregulation	–	–	Promotes metastasis	[91]
Breast cancer	Homozygous deletion	–	–	Associated with greater survival of breast cancer patients	[90]
Chronic lymphocytic leukaemia (CLL)	1 Mb deletion	–	–	Associated with CLL progression	[93]
Melanoma, lung adenocarcinoma, colorectal carcinoma	Upregulation	Inhibition	–	Higher expression of SETDB2 associated with adaptive resistance	[15]

This apparent context-dependent role for SETDB2 in tumour development is likely due to its regulation of genomic stability and/or H3K9me3-mediated silencing. Cancer cells in proliferative phases are largely heterogeneous and have varying needs for different cancer types, which could explain the differences in SETDB1 and SETDB2 expression for the progression of different cancers. However, they appear to have more tightly regulated roles in the context of adaptive resistance. Accumulating research shows that in response to therapies that target proliferative cells such as MAPK or EGFR inhibitors, cancer cells that initially responded well eventually display reduced sensitivity to treatment by altering their transcriptional profile and subsequent physiology through coordination of H3K9me3 levels [5, 14, 15]. During exposure to chemotherapeutics or targeted therapies, we observed the consistent upregulation of SETDB1 and SETDB2 in melanoma, lung and colon cancer-derived cell lines, which indicates a common role for both proteins following drug exposure [15]. Considering many antineoplastic treatments operate by inhibiting proliferation and inducing apoptosis via replicative stress such as DNA damage agents, MAPK inhibitors and cell cycle inhibitors, it is plausible that tumour cells utilise an increase in SETDB1 and SETDB2 to mitigate the effects of genomic instability and subsequent cell death in drug-exposed cells.

Our lab and others have also observed consistent IFN pathway enrichment following exposure to cytotoxic treatments [14, 15]. SETDB2 is reported to be an interferon-stimulated gene (ISG) downstream of type 1 IFN signalling and responsible for the attenuation of pro-inflammatory and antiviral genes induced by type I IFNs and the transcription factor NFkB [38, 95]. NFkB can be activated by DNA damage as a typical effect of chemotherapy or replicative stress to stimulate IFN production and suppress proliferation and apoptosis in the initial stress response [53, 96]. However, prolonged or heightened IFN signalling eventually causes increased cell death [97]. It is therefore vital for tumour cells to tightly regulate IFN signalling to survive treatment, which is plausibly mediated through the attenuation of ISGs by SETDB1/2 following an initial burst of inflammation and dedifferentiation that promotes their activation. It is reasonable that through SETDB1/2 mechanisms, cells have evolved a reliable and robust negative feedback response to stress-mediated IFNs that can promote a resilient, reversible phenotype through epigenetic, time-dependent and homeostatic coordination of IFN signalling.

SETDB2’s ability to diminish type I IFN signalling is a response mechanism that has also been observed for SETDB1 through repression of ISGs when bound with its recruiting cofactor KAP1 [98]. Both SETDB1 and SETDB2 proteins have shown the ability to inhibit IFN signalling as well as be potentially stimulated by it, although their modes of induction differ. Unlike SETDB2’s direct induction by activated STAT1, SETDB1 is induced indirectly by IFNβ through non-canonical Wnt5A signalling in a CaMKII–TAK1–TAB2–NLK cascade [99]. Treatment with recombinant human IFNβ showed a fivefold increase in Wnt5A mRNA levels in human ovarian cancer cells [100]. SETDB1 has also been shown to stimulate the Wnt pathway in NSCLC which could imply positive autoregulation; however, this study only looked at canonical Wnt-β-catenin signalling [101].

**Fig. 3:**
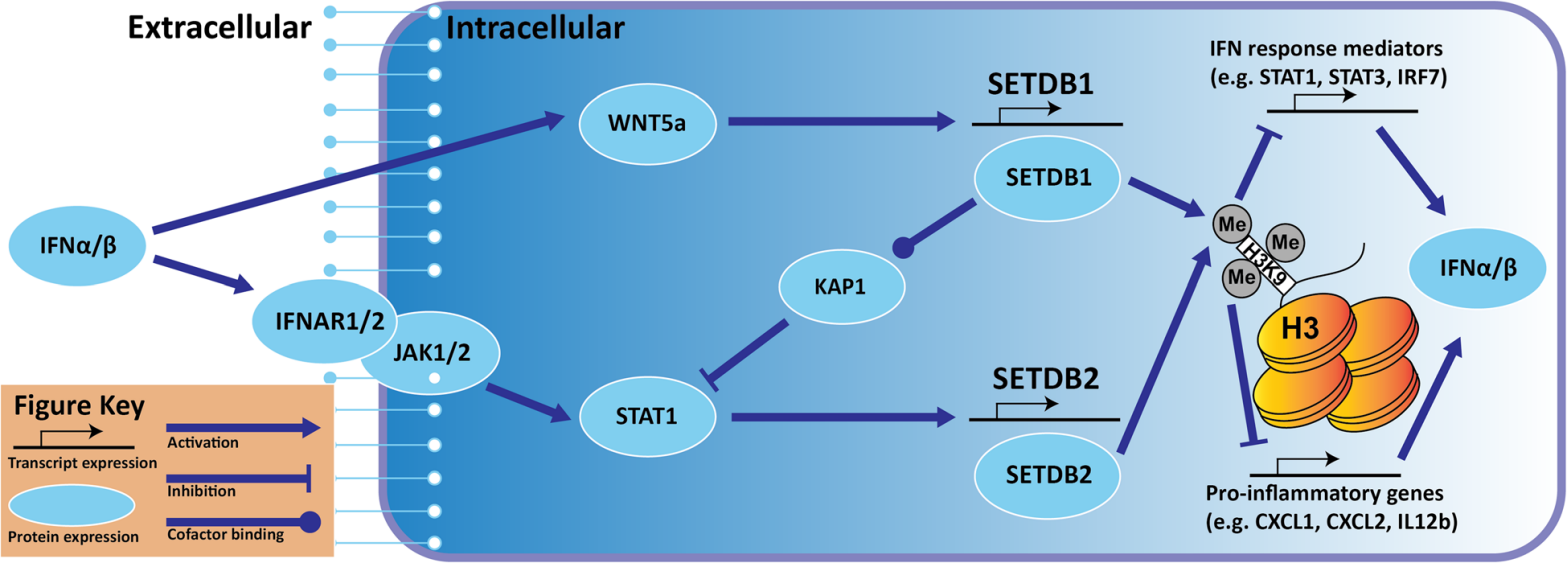
A hypothetical mechanisms for type I IFN-mediated induction of SETDB1 and SETDB2 by STAT1 and Wnt5a respectively. Feedback attenuation proposed through inhibition of pro-IFN signalling cytokines and KAP1-SETDB1 interactions. Key at bottom

It is important to note that SETDB2 induction by STAT1 was performed in healthy macrophages, but the existence of IFN-mediated induction of SETDB1 or SETDB2 in cancer cells is still unproven; however, it is reasonable that these immune-responsive and epigenetic mechanisms are highly conserved across cell types. The above studies outline a stepwise process led by IFN-mediated induction of SETDB1 and SETDB2 followed by recruitment and repression at ISG promoters to inhibit inflammation and contribute to the slow-cycling phenotype (Fig. 3). Whereas SETDB2 is directly downstream of activated STAT1 as shown by Schliehe et al., SETDB1 may instead be downstream of the type 1 IFN pathway through Wnt5a signalling as an intermediary as suggested above. Their inhibition of ISGs is then proposed to autoregulate the production of type 1 IFNs in order to tightly regulate the effects of IFN signalling. The extent in which this putative mechanism plays a part in promoting phenotypic switching to survive treatment, particularly in tumour cells with hyperactive growth pathways, is still being determined; however, our current understanding is growing rapidly. For example, the modulation of IFN signalling following BRAF inhibitor treatment in melanoma shown in Katlinskaya et al. demonstrates that fast-growing cells and slow-cycling cells have distinct levels of IFN signalling that can greatly impact their overall phenotype [33].

## Conclusions

**Fig. 4:**
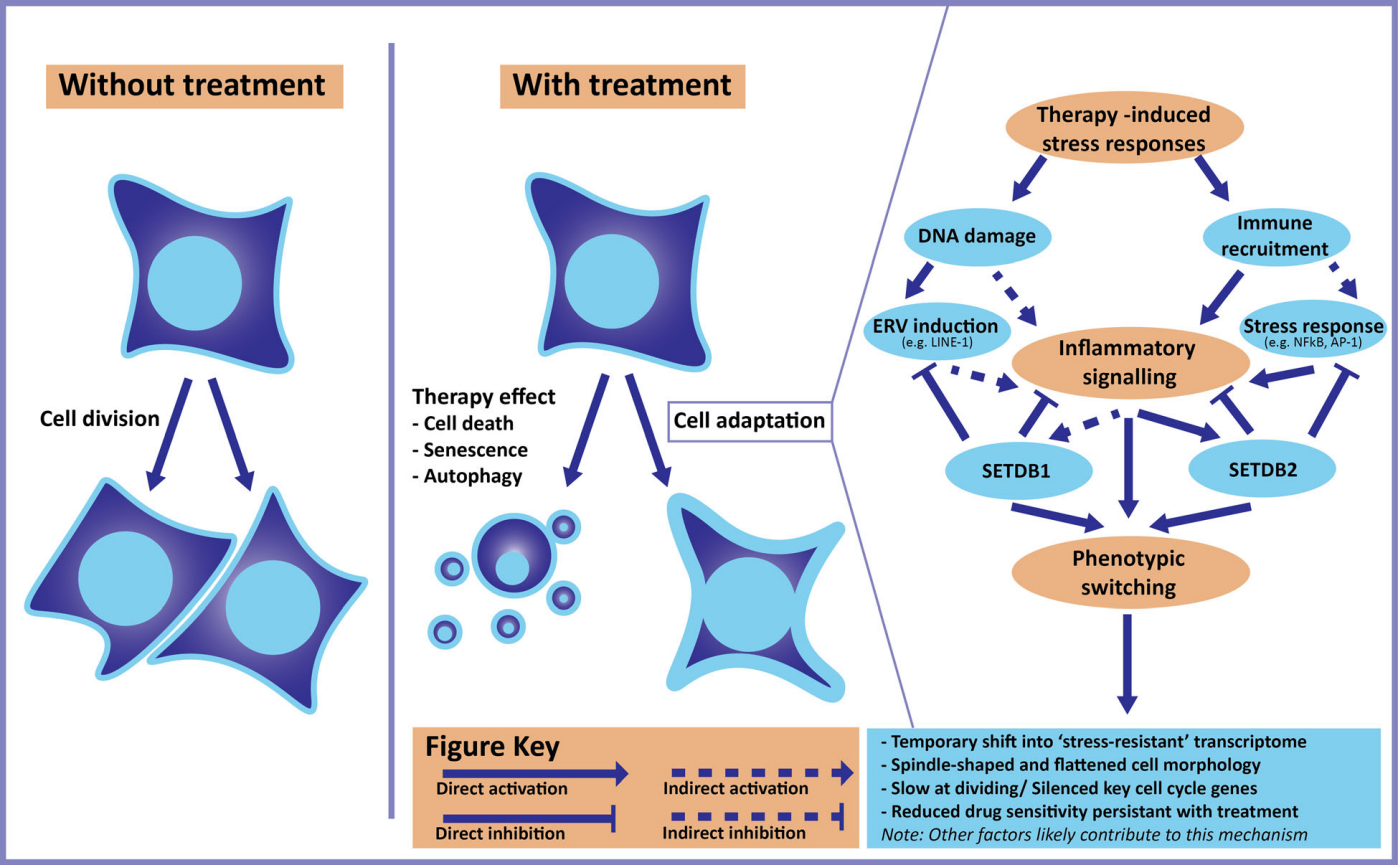
A speculative model for therapy-induced transcriptional remodelling. Detailed information of this model gathered using literature reviewed in the main text. Key at bottom

These studies elucidate that the frequently reported reversible resistance seen in treated tumour populations such as BRAF mutant melanoma or EGFR mutant NSCLC may be an attribute of a common resistance mechanism achieved through phenotypic switching and alteration of epigenetic landscapes. This putative mechanism is stimulated by treatment and the stress-induced expression of LINE-1 to upregulate IFN response genes and inflammatory signalling, subsequently promoting cell cycle arrest and dedifferentiation alongside upregulation of histone modifiers such as SETDB1 and SETDB2 (Fig. 4). These in turn lead to global epigenetic remodelling through histone modifications such as H3K9me3 that can inhibit the initial increase in LINE-1 and coordinate a common transcriptome of surviving tumour cells. Continuous treatment induces constant changes in treated cells marked by a distinct transcriptional trajectory associated with differing levels of differentiation, eventually reactivating or rewiring mitogenic pathways to resume high levels of proliferation while maintaining therapeutic resistance. The determination of whether this possible resistance mechanism is facilitating the development of acquired drug resistance in cancer populations will require further studies.

An understanding of reversible resistance is only beginning to emerge in the literature and the details and truth of the mechanism outlined here remains to be seen. Nonetheless, the collective attributes of the resistant cells across these studies cannot be ignored, and the central role that epigenetic modifiers such as SETDB1, SETDB2 and their product H3K9me3 share in developing these attributes is now finally becoming coherent. Eventually, continued investigation will yield valuable insights about cell biology, transcriptional survival mechanisms and potentially the generation of specific inhibitors of SETDB1 and SETDB2 to trial alongside current therapies (such as immuno- and targeted therapy) to maximise the efficacy, length and clinical benefit of modern cancer treatments.

## Abbreviations

ATAC-seq: Assay for Transposase-Accessible Chromatin using sequencing
ChIP-seq: Chromatin ImmunoPrecipitation sequencing
DTEP/DTPP: Drug-tolerant expanding/proliferating persister
DTP: Drug-tolerant persister
FISH: Fluorescent in situ hybridisation
H3K27ac: Acetylated lysine 27 of histone 3
H3K27me3: Trimethylated lysine 27 of histone 3
H3K9me3: Trimethylated lysine 9 of histone 3
ICB: Immune checkpoint blockade
IDTC: Induced drug-tolerant cell
IFN: Interferon
LINE: Long interspersed repetitive element
MAPK: Mitogen-associated protein kinase
NSCLC: Non-small-cell lung cancer
SETDB1/2: SET domain bifurcated 1/2
